# Leadership for a healthy work environment – a question about who, what and how

**DOI:** 10.1108/LHS-06-2020-0041

**Published:** 2021-12-02

**Authors:** Åsa Vidman, Annika Strömberg

**Affiliations:** 1 Department of Social Work and Criminology, University of Gävle, Gavle, Sweden; 2 Faculty of Health and Occupational Studies, University of Gävle, Gavle, Sweden

**Keywords:** Health, Leadership, Work environment, Elderly care, Qualitative content analysis

## Abstract

**Purpose:**

Recruiting and retaining staff to work with elderly people in social care is a global issue. The quality of leadership is considered important because it influences employees’ job satisfaction, job turnover and health. This paper aims to identify leadership that employees in residential elderly care facilities in Sweden consider as contributing towards a healthy work environment.

**Design/methodology/approach:**

The authors interviewed 14 persons employed in facilities organized in different ways. The data from these interviews was analysed using qualitative content analysis..

**Findings:**

The results showed that the employees felt that their health partly depended on the attributes that leaders possessed, what leaders do and how leaders do it. This study confirms that leadership influences the perception of a healthy workplace. It also shows that questions about leadership are complex.

**Originality/value:**

Research about factors that increase health risks is wide-ranging; however, research that examines factors that promote health, especially how leadership influences employees’ well-being, is not as comprehensive.

## Introduction

According to the World Health Organization (WHO) (2015), the world’s population aged 80 years or over will increase, and therefore, the number of elderly persons requiring support and care will increase (WHO, 2016). Thus, the recruitment and retention of staff in social care working with elderly are both important international issues (Chester *et al.*, 2014; Read and Fenge, 2019; Stone, 2016; Wong *et al.*, 2014). Because of the increasing elderly population, the Swedish Government estimates that the demand for employees within this sector will increase with 65% by the year 2035 (Skr, 2017/18:280).

In Sweden, residential care facilities often consist of several units that each house 8-12 residents (Szebehely, 2016). The units are organized to simulate a home environment, not a hospital ward, and there is little differentiation between the care workers. Furthermore, Szebehely (2016) points out that because the Swedish welfare state spends more than most other countries on elderly care (measured as a percentage of the gross domestic product (GDP)), the number of employees in the sector is comparatively high.

Health and social care workers in Sweden is one of the largest groups of employees (Statistics Sweden, 2020), and they have one of the highest rates of days on sick leave per employee (Swedish Social Insurance Agency, 2018). The Swedish Work Environment Authority (2018) shows that social and organizational issues account for about 58% of the sick leave due to work-related illnesses among the staff in residential homes for the elderly. The high number of employees and the rate of sick leave in the sector highlight the need for enhanced knowledge about the social and organizational work environments.

In elderly care, researchers consider the quality of leadership to be an important resource in a healthy organization (cf. Arnold, 2017; Keisu *et al.*, 2018). Furthermore, leadership in this particular sector influences employees’ job satisfaction (Westerberg and Tafvelin, 2014), job turnover (Jeon *et al.*, 2010), perception of empowerment (Hagerman *et al.*, 2017) and health (Ljungblad *et al.*, 2014). Although it is clear that leadership can help minimize sick leave for employees (Clausen *et al.*, 2012), there are no clear theories that explain and identify what constitutes this kind of leadership.

Research about illness and sick leave usually focuses on hazards in the work environment (Lindberg and Vingård, 2012; Swedish Agency for Work Environment Expertise, 2020a) rather than on factors that promote a healthy work environment. Although the rate of sick leave is high in elderly care, some workers in this sector maintain their health and enjoy their work (Swedish Social Insurance Agency, 2020). Moreover, while there are several studies about the importance of leadership when it comes to employees well-being (Swedish Agency for Work Environment Expertise, 2020b), there are few empirical studies that examine how employees understand, construct and give meaning to leadership in elderly care. The present article aims to identify leadership that employees within residential elderly care facilities in Sweden consider as contributing towards a healthy work environment. When discussing leadership in this article, it includes both managerial and leadership functions that are included in formal managers’ roles in elderly care.

## Perspectives on leadership

The field of leadership has been a fast-growing area of research and of great interest for practice since the beginning of the 20th century. The historical development of the field shows different areas of focus (Dinh *et al.*, 2014; Day *et al.*, 2014). Traditionally, the field has centred on individual leaders and their traits and abilities. Recently, researchers claimed that leadership is about interaction and depends on the context and situation, i.e. not merely about the leaders’ abilities and personality (Aalateeg, 2017).

Uhl-Bien (2006) discusses the difference between an entity approach and a relational approach when studying leadership. The *entity perspective* assumes the individuals are independent entities, and such an approach is consistent with an epistemology that values objective truth. The focus is on leadership styles and the behaviour in relationships among leaders and followers – in this case, employees. Individual perceptions, cognition, attributes and behaviour influence relationships among leaders and employees. The *relational perspective* views knowledge as socially constructed, and not as individuals’ mind-sets. Leadership, according to the relational view, is the processes by which social order is constructed and changed. In this perspective, individuals are not separable. Relations are made in communication processes, and leadership is a social reality, emergent and inseparable from context. “Entity perspectives (e.g., relationship-based leadership) emphasize the importance of interpersonal relationships, while relational perspectives (e.g., relational constructionism) emphasize the importance of ‘relating’ and relatedness (i.e., the processes and condition of being in relation to others and the larger social system in constructing the meaning and reality of leadership).” (Uhl-Bien, 2006, p. 664). Crevani *et al.* (2010) point out that “[o]ne common theme is that current leadership research is built on a taken-for-granted individualism that must be articulated and challenged” (p. 79). They think that we have to “try to redefine leadership in terms of processes and practices organized by people in interaction” (p. 78).

A common way to categorize leadership models is as either task- or relation-orientated leadership (McCleskey, 2014). Research shows that different leadership models have different effects on performance, job satisfaction and health (McCleskey, 2014). For example, task-orientated leadership has shown effects on performance, while relation-orientation leadership supports job satisfaction (Fernandez, 2008). Relation-orientated leadership has also shown positive effects on employees’ well-being (Skakon *et al.*, 2010; Schmidt *et al.*, 2014). One form of relational leadership is transformational leadership, a style often adopted in the health-care sector (Elshout *et al.*, 2013; Tafvelin *et al.*, 2018).

A leader using a transformational leadership style acts as a role model, adds meaning and challenge to subordinates’ work, encourages subordinates to be creative and approach problems in new ways, pays attention to the individual subordinate’s needs and provides coaching and mentoring (Skakon *et al.*, 2010). Transformational leadership has been shown to have a positive impact on job satisfaction and well-being (Sow *et al.*, 2017; Morsiani *et al.*, 2016; Sudha *et al.*, 2016) as well as on the quality of care in the health sector (Havig, 2011; Westerberg and Tafvelin, 2014).

As stated above, studies on leadership often divide leadership models into different categories that reflect either one approach or the other. Research on leadership within the health-care sector has highlighted the sector-specific situation and the need for theories customized for this specific context. In addition, research in this sector often points out the demanding situation for leaders who must simultaneously handle both professional and managerial needs and expectations (Ekholm, 2012; Hasenfeld, 2015; Vito, 2019). Leaders must deal with the different and sometimes contradictory logics of the professional and managerial sides, and this often triggers continuous reorientation of their daily work organization (cf. Kristiansen *et al.*, 2015). Lundberg *et al.* (2016) suggest that different contexts for leadership performance require different leadership characteristics to create, support and maintain a sound psychological work environment:

By recognising the differences in contextual structures and recruiting leaders with the characteristics that are desirable for the specific context, there is a greater potential for creating a positive psychosocial work environment in old age care (Lundberg *et al.*, 2016, p. 52).

In Sweden, residential elderly care facilities are a part of the social services (cf. Sweden.se, 2018). Social work research highlights the problem that leadership in this area is difficult to define (Holosko, 2009; Lawler, 2007; Sullivan, 2016). Peters (2018) considers it a problem that the growing number of studies on leadership in social work use theories of leadership developed for corporate entities, which have different goals and processes than do social work organizations. The results from his study show the interdependent relation between the organizational, relational and individual levels with regard to leadership. Peters (2018) suggests that leadership in social work can be defined as:

A collection of organizational, relational and individual behaviors that effect positive change in order to address client and societal challenges through emotional competence and the full acceptance, validation and trust of all individuals as capable human beings (p. 40).

## Methods

The data analyzed in this article were generated in qualitative interviews and were extracted from a larger project that aimed to answer what, from the staff’s perspective, in the work environment promoted the health of employees in elderly care. After conducting the interviews, we first sorted the data in different data sets (Braun and Clarke, 2006). This article focuses on one of the data sets – leadership that contributes towards a healthy work environment from the perspective of staff employed in residential care facilities for elderly people in Sweden. Inspired by researchers like Rojiani (1994), this study focused on understanding the employees’ understanding, constructs and the meaning they give towards leadership.

To get a broad view, we conducted interviews with participants from different working contexts. To obtain this purposive sampling, we contacted managers of residential care facilities in central Sweden that differed in size and operational mode. In Sweden, the employer pays for the first two weeks of a period of sick leave; after this, the Swedish Social Insurance Agency is responsible for the allowance. Therefore, we instructed the managers that we wanted to contact employees who had not been on sick leave for more than two weeks during the past year. In two facilities, the managers declined to participate due to re-organization, and in two additional facilities, the managers gave no reason for not wanting to participate. In five residential care facilities, the manager helped us to contact the employees. Three of these facilities were operated by a municipality, one was a cooperative and a joint-stock company ran one. The number of apartments in the facilities varied between 9 and 120. The facilities included units for somatic disorders, for persons with dementia and for short-stay residence.

The employees who participated in the interviews comprised 13 women and one man, with an age range of 31-65 years. Most of the employees were born in Sweden (two were immigrants). One participant had a university degree, and the others had an upper secondary-level education. Demographic information was collected by direct questions posed to the participants during the interviews. All of the participants were informed both orally and in writing about the aims of the study, that their identities would remain anonymous, that their participation was voluntary and that their participation would not affect their employment.

Both authors conducted the interviews, which took place in the facility where the participant worked and lasted between 40 min and 1 h 45 min. Interviews were conducted individually with 11 employees; at one facility, the manager had asked three persons to participate in the interview at the same time. During the interviews, we strived for narrative-style responses. Just as in teller-focused interviewing as described by Hydén (2014), we concentrated more on listening than asking questions; the interviews were almost totally unstructured (Bryman, 2016). We did, however, use a script that contained the topics to be discussed (Brinkmann and Kvale, 2015) as follows: why do you work in this profession, the meaning of work, what is needed for job satisfaction, what promotes well-being and health, leadership importance and possibilities and possibilities and conditions in the elderly care sector. The interview questions were open-ended, and the interviews began with a question like “Can you please tell us how it came about that you are working here?” Prompts such as “please tell me more?” or “what does that mean for you?” were used to clarify the working experience. The interviews were recorded and transcribed verbatim by the first author.

The transcribed interviews were analyzed using qualitative content analysis – “a research technique for making replicable and valid inferences from texts (or other meaningful matter) to their contexts of their use” (Krippendorff, 2004, p. 18). Sometimes, content analysis is considered to be about counting (Floersch *et al.*, 2010), but qualitative content analysis can also be about the latent meaning in a text (Wilhelmsson Göstas *et al.*, 2012). Thus, content analysis can be manifest when describing the content, and latent when interpreting the meaning (Berg and Lune, 2012). It can be described as a “careful, detailed, systematic examination and interpretation of a particular body of material in an effort to identify patterns, themes, biases, and meanings” (Berg and Lune, 2012, p. 349).

The analysis process was influenced by Graneheim and Lundman (2004) and Graneheim *et al.* (2017) and was performed in the following ways: to get a sense of the material as a whole, the first author read the text and the second author both listened to the recordings and read the texts; the first author created a first analysis scheme with meaning units, condensed meaning units and codes; both authors met frequently to compare and discuss codes, sub-themes and themes; the condensed meaning units were labelled with a code and abstracted to a sub-theme. We compared condensed meaning units and sub-themes and themes with each other until we agreed on how to sort the data. After this, we arrived at an agreement about the themes and the main theme, i.e. the latent meaning. Examples are presented in Table 1 of the process from meaning unit to theme.

### Methodological considerations

The trustworthiness of the study is discussed using the concepts of credibility, dependability and transferability (Graneheim and Lundman, 2004; Graneheim *et al.*, 2017). With an intent to establish *credibility*, we contacted managers from different-sized facilities and in different modes of operation to get in contact with interviewees. Even if the research focus in the larger project was about the work environment as a whole, whether the managers chose staff who had a positive view of the leader can be questioned. This may have affected the results in a way that the chosen employees highlighted the importance of managers more so than other employees would have. During the interviews, the employees did not give too much attention to the leadership, and they emphasized other aspects in the work environment.

The interviewees were both male and female, had a wide range of ages and came from different countries, which altogether could give various experiences. Even if the number of interviewees was small, the interviews provided robust data. In the last interviews, there was not much new information. In this sense, we felt that we had reached saturation.

When deciding how to sort meaning units, codes and categories, the process described above with both authors involved addressed the question of credibility as well as that of *dependability*. Dependability is also about the authors’ pre-understandings. The authors’ pre-understanding was vague about the staffs’ view of leadership that contributes towards a healthy work environment in these particular settings. We were aware of how elderly care facilities worked in general, but we had not done studies in this context before.

The selection of interviewees described above together with the broad view of the context described in the Introduction is a means of appropriating *transferability*. The assumption is that the reader can judge if the findings can be transferred to other contexts.

## Findings

The analysis shows three themes for leadership that the employees consider contribute towards a healthy work environment – what leaders do, how they do it and who they are. The main theme that emerged through the analysis was that employees prefer responsible leaders with confidence and the ability to manage change through trustful interaction. The findings are presented in Table 2 and described below.

### What to do

When talking about what the leaders ought to do to support a healthy work environment, the employees expressed that leaders should both maintain order and solve problems.

#### Maintain order.

The leader has to assign responsibilities by delegating tasks and making it clear who is responsible for what tasks. The participants also wanted the leader to be competent at handling paperwork and at writing. The leader is also responsible for recruiting staff, and when the leader is careful about whom to employ, this gives a sense of being important. One of the interviewees expressed this in the quotation below:

Well, I think that [the leader] as a leader is very careful about whom she employs. And it is the same when taking on a temporary substitute and everything you feel confident that she […] wants it to be in order (Anna).

The leader has an important task to pass information between the employees and the central administration. In addition, it is important that the leader explains and makes sense of the decisions handed down from politicians and the central administration. One way of conveying this information is through regular staff meetings. In addition, leaders can provide information when meeting with the staff on the ward; these interactions often occur in the morning. According to participants, these meetings make them feel secure in knowing that these interactions give them the chance to provide and receive crucial information. The participants appreciated a leader who was approachable and available, via phone or in person, for individual consultations.

#### Problem-solving.

Solving problems is about taking part in caring activities. If the leader lends a hand in an extraordinary situation, this is considered a sign that he or she does whatever is needed to shield and support the staff while performing the tasks required for the job.

The employees are aware of the economic situation and appreciate a leader who can use resources economically. However, they believe it is important that the leader does not hesitate to act when needed, even if the action involves spending money. Such expenditures may include providing the staff with a consultant when the job situation is especially hard or providing a massage chair to be used as a means to handle job stress.

But, first and foremost, employees express satisfaction when a leader takes responsibility for staffing and recruiting, especially when it requires providing a sufficient number of staff when the situation warrants extra help. In addition, the staff wanted leaders who are helpful when it comes to scheduling vacation or leave at short notice or who take the lead in helping staff when they have problems, as in the following excerpt:

We had it really hard. He [the son of an employee, who was a victim of school bullying] felt really bad when he went to school and I [the mother] had to be here to plan and fix. But then [the leader] is absolutely wonderful. I was not required to be here. “You go home and I'll get a replacement,” she said. “You go home. You should take care of your boy” […] “You can do that [make up hours] another time.” So she said, “Now you relax. Now you don’t even think about the job. I’ll fix it”. (Beatrice)

Clearly, the employee was moved by the leader’s concern for her and her son’s well-being beyond the workplace.

Furthermore, the leader should represent the facility in meetings with the central administration. When the employees feel confident that their leader is an advocate for their interests, a sense of trust is built between them and their leader.

### How to do it

The analysis shows that it is also important *how* the leaders do what they do. It is both about the necessity of trusting the employees and being trusted by the employees, a feeling created through open and respectful interaction between a leader and the staff.

#### Trusting the staff.

To feel confident about one’s own ability and performance, it is important to receive helpful feedback from the leader. Effective feedback requires a leader to identify an employee’s strengths as well as weaknesses. When the employees feel they can trust that the leader provides honest feedback, they also have a feeling that they are important and trusted.

Feedback is also connected to the leader’s reactions when the staff provides suggestions about how to do the work, which is exemplified with the following quotation from one of the interviewees:

She trusts us. She does. There is no problem in that way, but it is understood that she trusts that we do what we have to (Cecilia).

The interviewees also noted that it was important that the leader does not interfere in their daily work. They describe a leader as someone who implicitly relies on and trusts the employees to do what is expected. The leader does not oversee the staff, but instead says, “well, you fix this”.

#### Interaction between leader and the staff.

The interaction is about listening and talking as well as unspoken communication. To be seen by the leader is something important, according to the staff. It can be something such as being aware that someone is experiencing pain and then giving them support. Such awareness requires a leader to know the staff, their strengths and their weaknesses. One of the interviewees expressed this idea when she said the following:

I know she knows that I can. And what I can (Doris).

When she is seen, she has a sense of being valued, and because the leader sees her, the leader knows her ability and trusts her. Interaction is strongly related to being trusted.

As mentioned above, listening to the employees is something a leader should do. Employees express that they know that they can talk to the leader, both if they feeling bad or good about organizational matters. When talking about the organization, it is important that they can have a discussion with the leader without having a feeling of just complaining or whining. Dialogue is demanded by the staff when talking about how to manage the organization. A leader who gives instructions without listening to the employees’ point of view is considered a problem. At the same time, interviewees talked about the leader as a person who sets the norms for how to act at the facility. The interviewees talked about the importance of the leader setting the tone and developing a sense of “we”, solidarity and fair play.

In addition, the staff acknowledged the importance of a responsive leader when they express their needs. The staff desired leaders who make it safe to discuss their problems and who, when appropriate, help provide solutions to these problems. To maintain such an open and trusting climate, the leader has to be easily approachable, physically as well as socially and psychologically.

### Who to be

The leader’s personal characteristics, the *leader’s attributes*, are also seen as important. A leader should inspire confidence and be viewed as a person with integrity. A leader should keep confidences. At the same time, a leader should be straightforward, someone who communicates in a clear and straightforward way:

Yes, in my opinion, a good leader is supposed to be clear and say if there is something (Cecilia).

When an employee has problems with their caring work, they want advice from the leader. Therefore, if an employee has a problem carrying out a job, a leader should have the insight and knowledge about the caring work to provide help. Basically, the employees want their leaders to have a background in caring work. One of the interviewees said:

And she does not hesitate to come in and help us with daily work if needed (Eva).

Furthermore, the leader must have the ability to take action. A leader should make definite and timely decisions even when initiating a serious or difficult talk with an employee. The staff needs to know that the leader does not let things be if there are problems. At the same time, the leader should be flexible. Employees want their leader to have the ability to change their plans if something unexpected happens. Leaders are not to be constrained by rules and procedures; instead, they should adapt to the situation.

## Discussion

There are studies pointing at the impact that leadership in elderly care has on the hazards in the work environment. Those studies often take departure in special leadership theories or use questionnaires where the researchers have chosen what they thought was important. It is not that common with studies where the understanding, meaning and constructs the employees give to the phenomena are in focus. As a contribution to fill this gap, the present article illuminates leadership that employees within residential elderly care facilities in Sweden consider as contributing towards a healthy work environment. The study shows that leadership in this perspective cannot be reduced to a single leadership style or model. Instead, leadership that contributes to a healthy work environment is about the interplay between who, what and how. The main finding is that one feature of a healthy work environment is a *responsible leader with confidence and ability to manage change through trustful interaction.*

The interpretation of the interviews is summarized in three themes of leadership contributing towards a healthy work environment – what leaders do, how it is done and who the leaders are. Looking at the three themes one at a time reveals leadership through an entity perspective which assumes “relational processes as centered in individuals’ perceptions and cognitions” (Uhl-Bien, 2006, p. 655). It is clear that the interviewees describe leadership as connected to a single person with the leadership role and something that can be articulated and organized beforehand. In some way, the interviewees tell us about individual leaders and their traits and abilities (Aalateeg, 2017).

However, the themes also include descriptions about the processes, especially in the theme “how to do it”, where interaction and trust in the staff are important aspects. This view has similarities with the theories of relation-orientated leadership (McCleskey, 2014) and transformational leadership (Skakon *et al.*, 2010). Still, we found this to be an entity perspective where the processes can be identified and described with examples and labelled with keywords. In a way, there is a presumption that it is possible to find the best way of organizing the processes of interaction and trust, and “leadership in conditions of already being organized” are the primarily focus in the entity perspective (Uhl-Bien, 2006, p. 664).

To describe our summarized picture of the leaders as responsible, with confidence and ability to manage change via trustful interaction, the themes need something more to come alive, something that describes not just the leader but also the responsibilities and what goes on between the leader and their staff. For example, the interviewees felt safe with respect to their relationship with their leader. They felt safe about the fact that their leaders would act, interact, inform, provide feedback, support and point out the direction of the work and how to organize it in a professional way. Positive relationships with supervisors have proven important for how work is perceived (Smith and Shields, 2013). The interviewees expressed trust for the leader and safety in the relationship and the processes.

The interviewees believed their work environment was open and responsive, qualities they attributed to their leader. In addition, they seemed confident that their leader acted in the best interest of the organization, the workgroup and the individual. The employees took for granted that the leaders had good intentions, and that they expressed the sense of being part of a community, where the leader is the one setting the basic values of the group. These aspects of leadership are not possible to plan beforehand and can hardly be seen as an entity perspective. Instead, these aspects show the importance of a relational perspective where the interpretation of leadership depends on the context and social constructions of reality in a process of organizing (Uhl-Bien, 2006). This approach was articulated only implicitly in the employees’ comments that leadership contributing towards a healthy work environment is sometimes about not interrupting daily work or meddling too much. Rather, it is about being there when needed. Crevani *et al.* (2010) argues for leadership research to focus on processes, practices and interaction and emphasize the need of “new paradigms and perspectives in order to escape the problematic individualism” (p. 84). Even if this study does not solely focus on processes, it is interesting to notice that employees talk about the interaction and what in the processes was important.

In the present study, the interpretations show that the interviewees understood that leadership that contributes to a healthy work environment could be seen both from an entity perspective and a relational perspective, although they did not use this terminology. To describe leadership just from an entity perspective was not enough for the interviewees to paint a clear picture. They also provided stories about the relational parts and the processes in which leadership was constructed and reconstructed. The relations were described as depending on the context, problems in every day work and the people involved – both leaders and co-workers. In this sense, it is not about *either* an entity *or* a relational perspective; it is about *both* at the same time. From the interviewees’ point of view, leadership that contributes to a healthy work environment is created in the dynamic processes between the entities’ characteristics and the relational and procedural parts of social constructions in everyday work. These findings support Peters’ (2018) definition of leadership that includes organizational, relational and individual aspects.

The context for the present study is elderly care, and as previous research has shown, the human services sector is supposed to embrace values that enhance human dignity and to offer services that reduce suffering and social inequality (cf. Hasenfeld, 2015). Working with these issues entails strong emotional elements, especially in services where employees work in the clients’ homes and are supposed to be part of everyday life, as in elderly care. As Peters (2018) states “there is a strong emotional aspect – both negative and positive – in social work practice that does not exist to the same extent in the for-profit world.” (p. 32). This means that leadership is often closely connected to the professional logic, which is an inherent part of professionals’ practices (cf. Kristiansen *et al.*, 2015). In the residential elderly care facilities included in the present study, the leaders were in close proximity to operational issues and had offices physically close to daily work. This entails special preconditions and expectations with regard to leadership.

### Future research

Given that most of the previous research has focused on health risks rather than factors that promote a healthy work environment (Lindberg and Vingård, 2012; Swedish Agency for Work Environment Expertise, 2020a), the present study contributes knowledge that deserves further research. The article emanates from a larger project aimed at illuminating what employees in elderly care consider a healthy work environment. Leadership was one of the aspects that emerged during the interviews. Therefore, a study focused solely on leadership might deepen the knowledge. In such a study, it would be useful to include observations and interviews or conduct an ethnographic approach. This would enable analysis of leaders’ actions, employees’ interpretations and leaders’ explanations of these actions.

Our method presupposed that we could not know in advance what issues the interviewees would consider important. Therefore, the number of interviewees in the present study is limited. This is not necessarily a problem. Nevertheless, to broaden our knowledge about the topic, it would be useful to conduct a larger study using questionnaires with a focus on the three themes in the result of this study.

The development of theories of leadership is a fast-growing field. For future research, it would be interesting to see studies of leadership in elderly care taking the point of departure in new theories of leadership, e.g. servant leadership, authentic leadership or ethical leadership, and compare these with the results of the present study.

## Conclusions and implications

The findings from the present study further support results from previous research showing that leadership influences the perception of a healthy work environment (cf. Arnold, 2017; Keisu *et al.*, 2018). The findings also indicate that questions about leadership are complex and depend on context (Ekholm, 2012; Hasenfeld, 2015; Vito, 2019). This indicates that leadership is a complex field. The interviewees in the present study talked about leadership as not just connected to a person and specific characteristics but also, equally important, about what a leader is supposed to do and how it is done – characteristics that focus on the essence of action and interaction. In this study, we show that leadership contributing towards a healthy work environment from the interviewees’ point of view is not about either personality questions or procedural/relational subjects. It includes both aspects together and at the same time. Simultaneously, considering *who*, *what* and *how* forms opinions about leadership, which is constructed and reconstructed in the processes. Leadership in this sense is not easy to define, articulate or pinpoint. The feeling that the work environment is healthy is constituted in processes and the social flows of interaction.

To conclude, the present study implicates that, to improve recruitment and retention of staff in elderly care, it is important to be aware of both the context and the significance of leaders’ influence. The results point to issues that can be on relevance for the leaders to be aware of and can be one subject in the educations of leaders. In addition, the knowledge can be important when recruiting leaders to residential care facilities. The leaders’ impact on the work environment depends on who the individual leaders are, what they do and how they do it. Therefore, organizations should be aware that employees prefer responsible leaders with the confidence and ability to manage change through trustful interaction.

## Figures and Tables

**Table 1.** Examples of meaning units, condensed meaning units, codes, sub-themes and the theme

Meaning unit	Condensed meaning unit	Code	Sub-theme	Theme
Yes, but that you can have open dialogue like this with her about ideas and what to think and feel	Have open dialogue about ideas and what you think and feel	Making dialogue	Interaction between leader and staff	How to do it
But there have been people who felt bad for one or another reason like in their home; but yes, then I can call [the leader] and she listens	The leader listens when you are feeling bad	Listen to the employee		
Well it’s that you get this, sometimes we are told that we are capable and are doing a good job but at the same time we hear when it becomes something that is wrong or some shortcomings; and so yeah, I think we have received the support anyway	We are told that we are good and do a good job as well as hearing when something is amiss or wrong	Feedback from the leader	Trusting the staff	

**Table 2.** Main theme, themes, sub-themes and examples of codes

Main theme	Responsible leaders with confidence and ability to manage change through trustful interaction				
Themes	What to do		How to do it		Who to be
Sub-themes	Maintaining order	Problem-solving	Trusting the staff	Interaction between leader and staff	Leader’s attributes
Examples of codes	Administration related to staff Giving information Keeping regular meetings Being reachable Delegating tasks	Dealing with staffing and recruitment Taking part in caring activities Representing the facility Taking responsibility and acting for the staff’s well-being	Feedback from the leader Reliance on staff being capable	To be seen by the leader Making dialogue Being responsive Listening to the employee Approachable style	Being flexible Ability to take action Insight and knowledge about caring Inspiring confidence Straightforward
